# Verbal fluency: Effect of time on item generation

**DOI:** 10.1590/S1980-57642011DN05020008

**Published:** 2011

**Authors:** Mayra Jacuviske Venegas, Leticia Lessa Mansur

**Affiliations:** 1 Department of Physiotherapy, Speech Pathology and Occupational Therapy of Faculty of Medicine of the University of São Paulo, São Paulo SP, Brazil.

**Keywords:** verbal fluency, evaluation, cognition, language

## Abstract

The distribution of item generation/time in the performance of elderly on verbal fluency (VF) remains unknown.

**Objective:**

To analyze the number of items, their distribution and impact of the first quartile on the final test result.

**Methods:**

31 individuals performed the tests (average age=74 years; schooling=8.16 years).

**Results:**

The number of items produced in the first quartile differed from the other quartiles for both semantic and phonologic VF where 40% of items were produced in the first quartile. No effect of age was found and schooling influenced performance on the first and second quartiles of semantic VF and on the 1^st^, 2^nd^ and 3^rd^ quartiles of phonemic VF.

**Discussion:**

This study contributes with the finding that asymptotic levels are attained prior to the 30 seconds observed in other studies, being reached at the 15-second mark. Furthermore, schooling was found to be associated to the number of items produced in both the first and 2^nd^ quartiles for semantic VF, and in 1^st^, 2^nd^ and 3^rd^ quartiles for phonemic fluency.

**Conclusion:**

The schooling effect was noted both in semantic and executive aspects of VF. The brief form of the VF test may represent a promising tool for clinical evaluation.

## Abstract (pt)

Desconhece-se a distribuição da geração de itens/tempo, no desempenho de idosos na fluência verbal (FV).

**Objetivo:**

Analisar número de itens produzidos, sua distribuição e o impacto do primeiro quartil no resultado final da prova.

**Métodos:**

31 indivíduos (média de idade=74 anos; escolaridade média=8.16) realizaram testes.

**Resultados:**

O número de itens produzidos no primeiro quartil diferiu dos demais nas fluências semântica e fonológica; 40% dos itens foi produzido no primeiro quartil. Não houve efeito de idade e a escolaridade influenciou o primeiro e segundo quartis da fluência semântica e os três primeiros quartis da fluência fonêmica.

**Discussão:**

O estudo mostra que a curva assintótica de desempenho nos quartis da FV pode ser notada antes dos 30 segundos observados em prévios estudos, já nos 15 segundos iniciais. Adicionalmente, nosso estudo mostra que o efeito de escolaridade está associado ao desempenho nos dois primeiros quartis na fluência semântica e aos três primeiros quartis na fluência fonêmica.

**Conclusão:**

O efeito de escolaridade foi notado em aspectos semânticos e executivos da FV. A forma abreviada da FV é interessante proposta para a avaliação clínica.

The verbal fluency (VF) test is one of the most frequently used instruments to evaluate cognition in its semantic and executive aspects. The instrument is based on verbal production in one-minute.^1^

The two most-frequently used variations of the test focus on semantic and phonemic criteria. In the former, words of the same semantic field are solicited (for example, animals) whereas in the latter, the individual is requested to evoke as many words with the same phoneme as possible (for example, words beginning with /a/). VF is a quick test which is easy to apply in a clinical context. It has been used to evaluate aphasics^2^ and neurodegenerative conditions.^3-6^

The Brazilian Academy of Neurology has included the VF among the tests suggested to aid in the diagnosis of dementias.^7,8^ Lopes et al.^9^ indicated that it is a useful tool for analyzing evolution and stage of Alzheimer Disease (AD). Besides diagnosing and follow-up, another study tested the possibility of employing the VF test to determine prognosis and survival.^10^

Apart from illness, numerous variables can interfere with VF where age,^11^ gender^12^ and education,^13,14^ number among the most studied.

In the studies cited above, item generation was analyzed during a one-minute period. Other authors have indicated the possibility of reducing this time interval, using 30 seconds instead of 60 seconds as the basis for analysis. The argument supporting this position is that the first quartiles alone can determine final test performance.^15^

The present study advances this line of reasoning and had the following aims:

[1] To verify if there is a difference between the results obtained during the first fifteen seconds and those of the other test quartiles for the verbal fluency test performed with semantic and phonemic criteria; and
[2] To verify the impact of production in the first quartile on the final result after one minute of item generation.

## METHODS

An observational descriptive cross-sectional study was conducted. The sample comprised 31 elderly individuals (19 female and 12 male) aged older than 60 years, with no cognitive alterations and no neurological or psychiatric disease activity.

To exclude cognitive and language alterations, the subjects were submitted to the Mini-Mental State Exam,^16^ Clinical Dementia Rating scale (CDR),^17^ and the Questionnaire of Cognitive Change.^18^

The Project was approved by the Research Ethics Committee of the institution and all of those selected signed a free Term of Informed Consent (Register CEP-HU/USP: 100511 0 - SISNEP CAAE: 0034.0.198.000-10).

The evaluation was carried out individually in a silent environment by the same examiner (MJV) through a single session with each participant.

After excluding those elderly with cognitive alterations, personal data was obtained such as name, age, schooling, profession and a brief medical history of cognitive complaints was registered.

The VF tests were then applied. The semantic VF test was applied first. The individual was instructed to say as many names of animals as they could recall for a one-minute period. Next, the phonologic VF was applied, in which the individual was instructed to say as many words as possible beginning with the letters /f/, /a/ and /s/. Under both conditions (phonologic and semantic VF) the final punctuation of the repeated words was excluded.

The number of words emitted in the quartiles was computed (1-15 seconds, 16-30 seconds, 31-45 seconds and 46-60 seconds).

The scoring of the first 15 seconds in the semantic and phonologic Verbal Fluency was compared to the other quartiles of the one-minute period.

## Statistics

Non-parametric tests were employed in all comparisons. The Friedman Test was used to compare the performance between quartiles on Semantic Fluency (animals) and F-A-S tests. The significant differences (p<0.05) were discriminated by the Wilcoxon Test (adjusted by Bonferroni’s correction). Spearman’s Correlation was used to check for correlations between the first quartile, age and education.

Patient data are presented in descriptive form.

## Results

Thirty-one elderly individuals meeting the inclusion criteria were evaluated in this study.

The descriptive analysis of the variables age and schooling are shown in Table 1.

**Table 1:** Description of age, schooling and Mini-mental State Exam results

	Minimum	Maximum	Median	Mean (SD)
Age	61	88	76	74 (6.67)
Schooling	0	15	8	8.16 (5.27)
MMSE	23	30	28	27.58 (1.76)

Table 2 contains the percentage distribution of performance by quartile.

**Table 2:** Percentage distribution of items generated by quartile.

Quartile criteria	1^st^ n (%)	2^nd^ n (%)	3^rd^ n (%)	4^th^ n (%)
Animals	8 (47.64)	5 (24.34)	3 (18.45)	3 (14.18)
F	5 (42.45)	3 (25.41)	2 (17.31)	2 (14.80)
A	40.67	24.15	20.48	14.67
S	40.28	23.18	19.42	17.97

Table 3 shows the first quartile and the differences relative to the other quartiles.

**Table 3:** Comparison between results found for quartiles.

Quartiles	Animals P value	/f/ P value	/a/ P value	/s/ P value
1 × 2	<0.001	<0.001	<0.001	<0.001
1 × 3	<0.001	<0.001	<0.001	<0.001
1 × 4	<0.001	<0.001	<0.001	<0.001
2 × 3	0.006	0.002	0.165	0.054
2 × 4	<0.001	0.001	0.003	0.038
3 × 4	0.280	0.325	0.038	0.537

Table 4 indicates the correlation between schooling and the quartiles.

**Table 4:** Correlation between age and schooling and first quartile of semantic fluency (animals) and phonemic (FAS) tests.

Pairs of variables	Quartiles	RS	p
Age × Vf semantic	1^st^ q 2^nd^ q 3^rd^ q 4^th^ q Total	-0.162 -0.247 0.106 0.102 -0.093	0.382 0.180 0.569 0.585 0.615
Schooling × Vf semantic	1^st^ q 2^nd^ q 3^rd^ q 4^th^ q Total	0.588 0.438 -0.112 0.026 0.433	<0.001 0.013 0.546 0.886 0.014
Age × Vf phonemic (FAS)	1^st^ q 2^nd^ q 3^rd^ q 4^th^ q Total	-0.046 -0.257 -0.307 -0.172 -0.237	0.802 0.161 0.092 0.354 0.198
Schooling × VF phonemic (FAS)	1^st^ q 2^nd^ q 3^rd^ q 4^th^ q Total	0.366 0.402 0.452 0.323 0.468	0.042 0.024 0.010 0.075 0.007

VF: verbal fluency; q: quartiles; rs: Spearman's coefficient.

The graph displays the number of items in each quartile.

**Graph:**
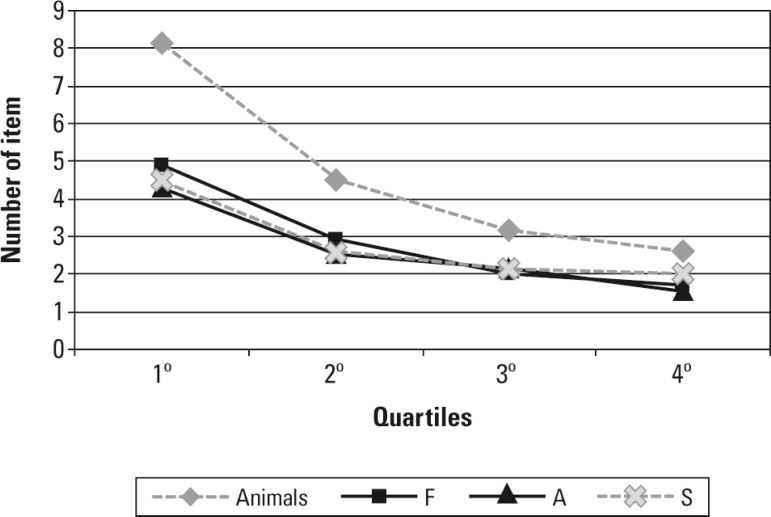
Production of items by quartile.

## Discussion

The present study detected differences between the results of the first 15 seconds compared with the other quartiles in the semantic and phonologic verbal fluency tests. Approximately half of the total items were produced in the first quartile. This difference had a significant impact on the total test score. Furthermore, a descending curve of word production was observed over the 60-second test period.

Besides corroborating the findings of Fernaeus et al.,^15^ regarding the descending curve of verbal production over time, as evidenced in the graph, the present study contributes with the finding that asymptotic levels are attained prior to 30 seconds, being reached at the 15-second mark.

In the Fernaus^15^ study, participants had schooling of greater than 9 years and a median age of 64 years. The effect of age on semantic fluency has been consistently demonstrated, as has the effect of schooling on both fluencies.^13,14^ The authors assumed the idea that schooling has an important role in the organization, consolidation and extension of semantic networks, the basis of the configuration of the evoked groupings.

Furthermore, the present study demonstrated that the effect of schooling was associated to the number of items produced: 1^st^, 2^nd^ quartiles and total for semantic verbal fluency; 1^st^, 2^nd^, 3^rd^ quartiles and total for phonemic VF.

Scores within specific intervals have been recognized as possessing significant power for predicting diagnosis.^15^ This was especially valid for intervals 1, 2 and 3 (in the study where the intervals were divided into 10 seconds) in the diagnosis of AD and MCI. According to the same authors, the principal differentiation factors of AD, MCI and individuals without objective cognitive disorders, were encountered in the first 3 intervals, and the most marked signs of dementia were presented in the first half of the test.

Another study demonstrated that, in addition to differentiating normal individuals from those that suffered a stroke, the test was able to differentiate aphasics and non-aphasics in this subgroup.^19^

The differences among the quartiles have sparked discussion about the demands and possible specificities of cognitive recruitment. In the situation of successful VF, the first quartile is dedicated to the execution of a previous scheme supported by semantic memory, while the other quartiles are implicated in planning, adjusting, and monitoring the performance, in order to guarantee the generation of items and avoidance of repetitions and intrusions.

The present study verified the influence of schooling on semantic sketch resources (performance in 1^st^ quartile) and planning and monitoring (2^nd^, 3^rd^ quartiles). Semantic memory sketch planning as well as planning and monitoring were impacted by schooling.

Studies employing functional imaging have identified that VF induced by semantic as well as phonologic criteria are entirely related to the language areas, such as the inferior frontal gyrus. This area has also been identified as “specialized” in fluencies.^20^ In focal lesions, activation of the pre-frontal and dorsolateral and ventromedial cortices was demonstrated in verbal fluency.^20,21^ In addition to recruiting of executive functions, the activation of other areas related to semantic processing has also been observed.

Given the broad potential for clinical application of the VF, it is important to verify the behavior of healthy aged individuals on the test in order to obtain references and norms.

The VF test has been acknowledged for the quantity of information that it yields in the context of clinical application and research. In addition to providing data about naming, the VF test assists in the evaluation of semantic memory, attention and inhibition of relevant items. The efficacy of comparative application in two modalities is beneficial not only in terms of time saving through quick application but also by reducing the stress and performance demands placed on patients with cognitive limitations.

Future studies involving a larger sample of elderly with cognitive complaints, controlled for different schooling levels, are a natural continuation to this investigation.

Critical analysis of the results enables the following conclusions to be drawn:

A substantial difference exists between results of the first 15 seconds on the Semantic and Phonologic Verbal Fluency tests compared with those of the other quartiles.

Schooling influenced the results on the 1^st^ and 2^nd^ quartiles for semantic verbal fluency, and on the 1^st^, 2^nd^ and 3^rd^ quartiles for phonemic verbal fluency. The impact of schooling was noted in both semantic and executive aspects of the task.
